# {5,5′-Dihydr­oxy-2,2′-[*o*-phenyl­enebis­(nitrilo­methyl­idyne)]diphenolato}nickel(II) dihydrate

**DOI:** 10.1107/S1600536808026093

**Published:** 2008-08-16

**Authors:** Hoong-Kun Fun, Reza Kia, Valiollah Mirkhani, Hasan Zargoshi

**Affiliations:** a X-ray Crystallography Unit, School of Physics, Universiti Sains Malaysia, 11800 USM, Penang, Malaysia; b Chemistry Department, Isfahan University, Isfahan, 81746-73441, Iran

## Abstract

In the title complex, [Ni(C_20_H_14_N_2_O_4_)]·2H_2_O, the Ni^II^ ion is in an essentially square-planar geometry involving an N_2_O_2_ atom set of the tetra­dentate Schiff base ligand. The Ni atom lies on a crystallographic twofold rotation axis. The asymmetric unit contains one half-mol­ecule of the complex and a water mol­ecule. An inter­molecular O—H⋯O hydrogen bond forms a four-membered ring, producing an *R*_1_^2^(4) ring motif involving a bifurcated hydrogen bond to the phenolate O atoms of the complex mol­ecule. In the crystal structure, mol­ecules are linked by π–π stacking inter­actions, with centroid–centroid distances in the range 3.5750 (11)–3.7750 (11) Å. As a result of the twofold symmetry, the central benzene ring makes the same dihedral angle of 15.75 (9)° with the two outer benzene rings. The dihedral angle between the two hydroxy­phenyl rings is 13.16 (5)°. In the crystal structure, mol­ecules are linked into infinite one-dimensional chains by directed four-membered O—H⋯O—H inter­actions along the *c* axis and are further connected by C—H⋯O and π–π stacking into a three-dimensional network. An inter­esting feature of the crystal structure is the short Ni⋯O, O⋯O and N⋯N inter­actions which are shorter than the sum of the van der Waals radii of the relevant atoms. The crystal structure is stabilized by inter­molecular O—H⋯O and C—H⋯O hydrogen bonds and by π–π stacking inter­actions.

## Related literature

For bond-length data, see Allen *et al.* (1987 ▶). For hydrogen-bond motifs, see: Bernstein *et al.* (1995 ▶). For related structures, see, for example: Clark *et al.* (1968 ▶, 1969 ▶, 1970 ▶); Hodgson 1975 ▶. For applications and bioactivities, see, for example: Elmali *et al.* (2000 ▶); Blower (1998 ▶); Granovski *et al.* (1993 ▶); Li & Chang (1991 ▶); Shahrokhian *et al.* (2000 ▶); Fun & Kia (2008**a* ▶,b* ▶).
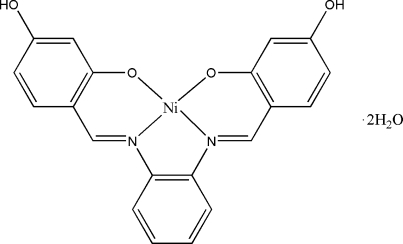


## Experimental

### 

#### Crystal data

[Ni(C_20_H_14_N_2_O_4_)]·2H_2_O
*M* *_r_* = 441.07
Monoclinic, 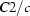

*a* = 10.9049 (2) Å
*b* = 17.6602 (3) Å
*c* = 9.0375 (3) Å
β = 101.150 (1)°
*V* = 1707.61 (7) Å^3^
*Z* = 4
Mo *K*α radiation
μ = 1.18 mm^−1^
*T* = 100.0 (1) K
0.35 × 0.12 × 0.11 mm

#### Data collection

Bruker SMART APEXII CCD area-detector diffractometer
Absorption correction: multi-scan (**SADABS**; Bruker, 2005 ▶) *T*_min_ = 0.683, *T*_max_ = 0.881
14574 measured reflections
3566 independent reflections
2388 reflections with *I* > 2σ(*I*)
*R*_int_ = 0.046

#### Refinement

*R*[*F* ^2^ > 2σ(*F* ^2^)] = 0.048
*wR*(*F* ^2^) = 0.120
*S* = 1.12
3566 reflections
136 parameters
H atoms treated by a mixture of independent and constrained refinement
Δρ_max_ = 0.61 e Å^−3^
Δρ_min_ = −0.73 e Å^−3^

### 

Data collection: *APEX2* (Bruker, 2005 ▶); cell refinement: *APEX2*; data reduction: *SAINT* (Bruker, 2005 ▶); program(s) used to solve structure: *SHELXTL* (Sheldrick, 2008 ▶); program(s) used to refine structure: *SHELXTL*; molecular graphics: *SHELXTL*; software used to prepare material for publication: *SHELXTL* and *PLATON* (Spek, 2003 ▶).

## Supplementary Material

Crystal structure: contains datablocks global, I. DOI: 10.1107/S1600536808026093/pk2114sup1.cif

Structure factors: contains datablocks I. DOI: 10.1107/S1600536808026093/pk2114Isup2.hkl

Additional supplementary materials:  crystallographic information; 3D view; checkCIF report

## Figures and Tables

**Table 1:** Selected interatomic distances (Å) *Cg*1, *Cg*2, *Cg*3, and *Cg*4 are the centroids of the Ni1/N1/C8/C8*A*/N1*A*, Ni1/O1/C1/C6/C7/N1, Ni1/O1*A*/C1*A*/C6*A*/C7*A*/N1*A* and C1–C6 rings, respectively.

*Cg*1⋯*Cg*4^i^	3.7364 (11)
*Cg*2⋯*Cg*2^i^	3.7380 (9)
*Cg*2⋯*Cg*3^ii^	3.7381 (9)
*Cg*3⋯*Cg*4^iii^	3.5766 (10)
*Cg*4⋯*Cg*4^iv^	3.7750 (11)
Ni1⋯O1*W*^v^	3.7635 (13)
O1⋯O1^v^	2.4319 (18)
N1⋯N1^v^	2.525 (2)

Symmetry codes: (i) 
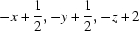
; (ii) 
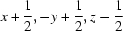
; (iii) 
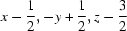
; (iv) 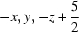; (v) 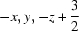.

**Table 2:** Hydrogen-bond geometry (Å, °)

*D*—H⋯*A*	*D*—H	H⋯*A*	*D*⋯*A*	*D*—H⋯*A*
O1*W*—H1*W*1⋯O1	0.88	2.40	3.0733 (18)	133
O1*W*—H1*W*1⋯O1^v^	0.88	1.97	2.8072 (19)	160
O1*W*—H2*W*1⋯O2^vi^	0.83	2.17	2.9985 (19)	173
C9—H9*A*⋯O2^vii^	0.93	2.60	3.394 (2)	144

Symmetry codes: (v) 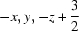; (vi) 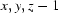; (vii) 
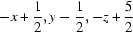
.

**Fig. 1.**
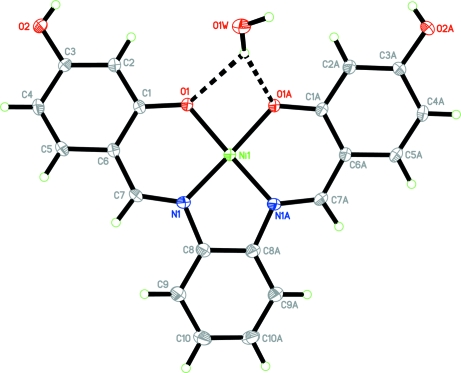
The molecular structure of the title compound, showing 50% probability displacement ellipsoids and the atomic numbering scheme. Intermolecular hydrogen bonds are drawn as dashed lines.

**Fig. 2.**
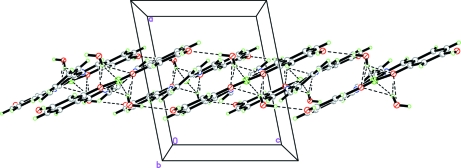
The crystal packing viewed down the b axis, showing one-dimensional extended chains involving the directed four membered O—H···O—H hydrogen bonds along the c axis. Intermolecular interactions are drawn as dashed lines.

## supplementary crystallographic information

### Comment

Schiff base complexes are some of the most important stereochemical models in transition metal coordination chemistry, with their ease of preparation and structural variations (Granovski *et al.*, 1993). Many of the reported structural investigations of these complexes are discussed in some details in a review (Hodgson, 1975). Metal derivatives of Schiff bases have been studied extensively, and Cu(II) and Ni(II) complexes play a major role in both synthetic and structural research (Elmali *et al.*, 2000; Blower, 1998; Fun & Kia, 2008*a,b*; Granovski *et al.*, 1993; Li & Chang, 1991; Shahrokhian *et al.*, 2000). Tetradentate Schiff base metal complexes may form *trans* or *cis* planar or tetrahedral structures (Elmali *et al.*, 2000).

In the title compound (Fig. 1), the Ni^II^ ion, is in an essentially square-planar geometry involving a N_2_O_2_ atom set of the tetradentate Schiff base ligand. The Ni atom lies on a crystallographic twofold rotation axis. An intermolecular O—H···O hydrogen bond forms a four-membered ring, producing an *R*^2^_1_(4) ring motif (Bernstein *et al.*, 1995). The bond lengths are within the normal ranges (Allen *et al.*, 1987). The asymmetric unit contains one-half of the molecule of the complex and a water molecule. The latter shows a bifurcated hydrogen bond which is connected to the phenolato oxygen atoms of the complex. The molecule is nearly planar, with a maximum deviation from the mean plane of 0.370 (2) Å for atom C9. As a result of the twofold symmetry, the central benzene ring makes the same dihedral angle of 15.75 (9)° with the two outer benzene rings.  The dihedral angle between the two hydroxy phenyl rings is 13.16 (5)°. In the crystal structure, (Fig. 2) molecules are linked into infinite one-dimensional chains by directed four-membered O—H···O—H interactions along the *c* axis and are furthered connected by C—H···O and π-π stacking into a three-dimensional network.

An interesting feature of the crystal structure is the short Ni···O, O···O, and N···N interactions (Table 1), which are shorter than the sum of the van der Waals radii of the relevant atoms. The short distances between the centroids of the five- and six-membered rings indicate the existence of the π-π interactions (Table 1). The crystal structure is stabilized by intermolecular O—H···O, C—H···O hydrogen bonds (Table 2) and π-π interactions.

### Experimental

A chloroform solution (40 ml) of the ligand (1 mmol, 354 mg) was added to a methanol solution (20 ml) of NiCl_2_.6H_2_O (1.05 mmol, 237 mg). The mixture was refluxed for 30 min and the resulting red precipitate was filtered, washed with cold ethanol and dried in air.  Single crystals suitable for *X*-ray analysis were obtained from a THF solution at RT.

### Refinement

The water H-atoms were located in a difference Fourier map and refined as riding on the parent atom with an isotropic displacement parameter of 1.5Ueq of the water oxygen. The hydroxyl H atoms were also located in a difference Fourier map and refined freely. The rest of the hydrogen atoms were positioned geometrically [C—H = 0.93 Å] and refined using a riding model.

### Crystal data

**Table:**

[Ni(C_20_H_14_N_2_O_4_)]·2H_2_O	*F*_000_ = 912
*M**_r_* = 441.07	*D*_x_ = 1.716 Mg m^−^^3^
Monoclinic, *C*2/*c*	Mo *K*α radiation λ = 0.71073 Å
Hall symbol: -C 2yc	Cell parameters from 3113 reflections
*a* = 10.9049 (2) Å	θ = 2.3–29.1º
*b* = 17.6602 (3) Å	µ = 1.18 mm^−^^1^
*c* = 9.0375 (3) Å	*T* = 100.0 (1) K
β = 101.150 (1)º	Block, red
*V* = 1707.61 (7) Å^3^	0.35 × 0.12 × 0.11 mm
*Z* = 4	

### Data collection

**Table:**

Bruker SMART APEXII CCD area-detector diffractometer	3566 independent reflections
Radiation source: fine-focus sealed tube	2388 reflections with *I* > 2σ(*I*)
Monochromator: graphite	*R*_int_ = 0.046
*T* = 100.0(1) K	θ_max_ = 34.3º
φ and ω scans	θ_min_ = 2.2º
Absorption correction: multi-scan(*SADABS*; Bruker, 2005)	*h* = −17→17
*T*_min_ = 0.683, *T*_max_ = 0.881	*k* = −23→27
14574 measured reflections	*l* = −14→14

### Refinement

**Table:**

Refinement on *F*^2^	Secondary atom site location: difference Fourier map
Least-squares matrix: full	Hydrogen site location: inferred from neighbouring sites
*R*[*F*^2^ > 2σ(*F*^2^)] = 0.048	H atoms treated by a mixture of independent and constrained refinement
*wR*(*F*^2^) = 0.121	*w* = 1/[σ^2^(*F*_o_^2^) + (0.0492*P*)^2^] where *P* = (*F*_o_^2^ + 2*F*_c_^2^)/3
*S* = 1.12	(Δ/σ)_max_ < 0.001
3566 reflections	Δρ_max_ = 0.61 e Å^−^^3^
136 parameters	Δρ_min_ = −0.73 e Å^−^^3^
Primary atom site location: structure-invariant direct methods	Extinction correction: none

### Special details

**Table:**

Experimental. The low-temperature data was collected with the Oxford Cryosystem Cobra low-temperature attachment
Geometry. All esds (except the esd in the dihedral angle between two l.s. planes) are estimated using the full covariance matrix. The cell esds are taken into account individually in the estimation of esds in distances, angles and torsion angles; correlations between esds in cell parameters are only used when they are defined by crystal symmetry. An approximate (isotropic) treatment of cell esds is used for estimating esds involving l.s. planes.
Refinement. Refinement of F^2^ against ALL reflections. The weighted R-factor wR and goodness of fit S are based on F^2^, conventional R-factors R are based on F, with F set to zero for negative F^2^. The threshold expression of F^2^ > 2sigma(F^2^) is used only for calculating R-factors(gt) etc. and is not relevant to the choice of reflections for refinement. R-factors based on F^2^ are statistically about twice as large as those based on F, and R- factors based on ALL data will be even larger.

### Fractional atomic coordinates and isotropic or equivalent isotropic displacement parameters (Å^2^)

**Table:**

	*x*	*y*	*z*	*U*_iso_*/*U*_eq_	
Ni1	0.0000	0.246535 (17)	0.7500	0.01493 (11)	
O1	0.04391 (12)	0.32501 (7)	0.88434 (14)	0.0174 (3)	
O2	0.18665 (13)	0.44913 (8)	1.35074 (15)	0.0215 (3)	
N1	0.06039 (14)	0.17016 (8)	0.88409 (17)	0.0152 (3)	
C1	0.09715 (16)	0.32009 (10)	1.0287 (2)	0.0159 (4)	
C2	0.11546 (17)	0.38691 (10)	1.1132 (2)	0.0175 (4)	
H2A	0.0908	0.4329	1.0668	0.021*	
C3	0.16965 (17)	0.38546 (10)	1.2647 (2)	0.0161 (4)	
C4	0.21006 (17)	0.31684 (11)	1.3364 (2)	0.0200 (4)	
H4A	0.2474	0.3160	1.4380	0.024*	
C5	0.1937 (2)	0.25113 (10)	1.2545 (2)	0.0192 (4)	
H5A	0.2218	0.2058	1.3015	0.023*	
C6	0.13518 (18)	0.25029 (10)	1.1002 (2)	0.0160 (3)	
C7	0.11768 (17)	0.17979 (10)	1.0251 (2)	0.0172 (4)	
H7A	0.1490	0.1369	1.0793	0.021*	
C8	0.03852 (17)	0.09645 (10)	0.8211 (2)	0.0181 (4)	
C9	0.08370 (18)	0.02822 (10)	0.8878 (2)	0.0206 (4)	
H9A	0.1404	0.0281	0.9790	0.025*	
C10	0.04344 (18)	−0.03919 (11)	0.8171 (2)	0.0232 (4)	
H10A	0.0748	−0.0849	0.8598	0.028*	
O1W	0.15531 (13)	0.42836 (7)	0.67062 (15)	0.0240 (3)	
H1W1	0.0971	0.3950	0.6774	0.036*	
H2W1	0.1630	0.4380	0.5829	0.036*	
H1O2	0.170 (2)	0.4836 (15)	1.304 (3)	0.047 (9)*	

### Atomic displacement parameters (Å^2^)

**Table:**

	*U*^11^	*U*^22^	*U*^33^	*U*^12^	*U*^13^	*U*^23^
Ni1	0.01935 (18)	0.01096 (18)	0.01281 (16)	0.000	−0.00102 (12)	0.000
O1	0.0240 (7)	0.0128 (6)	0.0126 (6)	0.0004 (5)	−0.0033 (5)	−0.0004 (5)
O2	0.0320 (8)	0.0146 (7)	0.0156 (6)	−0.0001 (6)	−0.0007 (6)	−0.0040 (6)
N1	0.0177 (7)	0.0109 (7)	0.0161 (7)	−0.0006 (6)	0.0012 (6)	−0.0003 (6)
C1	0.0189 (8)	0.0149 (9)	0.0129 (8)	0.0002 (7)	0.0002 (7)	0.0020 (7)
C2	0.0201 (9)	0.0156 (9)	0.0148 (8)	−0.0007 (7)	−0.0012 (7)	0.0009 (7)
C3	0.0192 (9)	0.0142 (9)	0.0145 (8)	−0.0009 (7)	0.0020 (7)	−0.0020 (7)
C4	0.0260 (10)	0.0209 (10)	0.0111 (8)	0.0015 (8)	−0.0011 (7)	0.0013 (7)
C5	0.0256 (10)	0.0168 (9)	0.0139 (8)	0.0010 (7)	0.0006 (7)	0.0047 (7)
C6	0.0189 (8)	0.0154 (9)	0.0126 (7)	−0.0004 (7)	0.0004 (6)	0.0010 (7)
C7	0.0215 (9)	0.0132 (9)	0.0158 (8)	0.0007 (7)	0.0009 (7)	0.0040 (7)
C8	0.0192 (9)	0.0151 (9)	0.0188 (9)	0.0006 (7)	0.0011 (7)	0.0016 (7)
C9	0.0224 (9)	0.0173 (9)	0.0207 (9)	0.0005 (8)	0.0007 (7)	0.0022 (8)
C10	0.0293 (11)	0.0153 (9)	0.0252 (10)	0.0014 (8)	0.0055 (8)	0.0039 (8)
O1W	0.0312 (8)	0.0175 (7)	0.0232 (7)	−0.0055 (6)	0.0050 (6)	0.0009 (6)

### Geometric parameters (Å, °)

**Table:**

Ni1—O1	1.8436 (12)	C4—C5	1.369 (3)
Ni1—O1^i^	1.8436 (12)	C4—H4A	0.9300
Ni1—N1^i^	1.8474 (15)	C5—C6	1.417 (3)
Ni1—N1	1.8474 (15)	C5—H5A	0.9300
O1—C1	1.324 (2)	C6—C7	1.414 (2)
O2—C3	1.359 (2)	C7—H7A	0.9300
O2—H1O2	0.74 (3)	C8—C8^i^	1.392 (4)
N1—C7	1.317 (2)	C8—C9	1.394 (2)
N1—C8	1.422 (2)	C9—C10	1.382 (3)
C1—C2	1.399 (2)	C9—H9A	0.9300
C1—C6	1.416 (2)	C10—C10^i^	1.387 (4)
C2—C3	1.383 (2)	C10—H10A	0.9300
C2—H2A	0.9300	O1W—H1W1	0.8771
C3—C4	1.404 (3)	O1W—H2W1	0.8309
			
Cg1···Cg4^ii^	3.7364 (11)	Cg4···Cg4^v^	3.7750 (11)
Cg2···Cg2^ii^	3.7380 (9)	Ni1···O1W^i^	3.7635 (13)
Cg2···Cg3^iii^	3.7381 (9)	O1···O1^i^	2.4319 (18)
Cg3···Cg4^iv^	3.5766 (10)	N1···N1^i^	2.525 (2)
			
O1—Ni1—O1^i^	82.53 (8)	C5—C4—H4A	120.5
O1—Ni1—N1^i^	174.29 (5)	C3—C4—H4A	120.5
O1^i^—Ni1—N1^i^	95.89 (7)	C4—C5—C6	121.82 (17)
O1—Ni1—N1	95.89 (7)	C4—C5—H5A	119.1
O1^i^—Ni1—N1	174.29 (6)	C6—C5—H5A	119.1
N1^i^—Ni1—N1	86.21 (9)	C7—C6—C1	123.15 (17)
C1—O1—Ni1	127.44 (11)	C7—C6—C5	118.38 (16)
C3—O2—H1O2	111 (2)	C1—C6—C5	118.47 (16)
C7—N1—C8	121.12 (15)	N1—C7—C6	124.97 (17)
C7—N1—Ni1	125.63 (13)	N1—C7—H7A	117.5
C8—N1—Ni1	113.24 (12)	C6—C7—H7A	117.5
O1—C1—C2	118.13 (16)	C8^i^—C8—C9	119.92 (11)
O1—C1—C6	122.74 (16)	C8^i^—C8—N1	113.20 (9)
C2—C1—C6	119.12 (17)	C9—C8—N1	126.87 (17)
C3—C2—C1	120.89 (17)	C10—C9—C8	119.37 (18)
C3—C2—H2A	119.6	C10—C9—H9A	120.3
C1—C2—H2A	119.6	C8—C9—H9A	120.3
O2—C3—C2	122.42 (17)	C9—C10—C10^i^	120.43 (11)
O2—C3—C4	117.00 (16)	C9—C10—H10A	119.8
C2—C3—C4	120.59 (17)	C10^i^—C10—H10A	119.8
C5—C4—C3	119.06 (17)	H1W1—O1W—H2W1	114.3
			
O1^i^—Ni1—O1—C1	−176.47 (18)	O1—C1—C6—C5	178.70 (17)
N1^i^—Ni1—N1—C7	−176.59 (19)	C2—C1—C6—C5	−1.7 (3)
O1—Ni1—N1—C8	177.60 (12)	C4—C5—C6—C7	−177.71 (18)
N1^i^—Ni1—N1—C8	2.93 (9)	C4—C5—C6—C1	2.4 (3)
Ni1—O1—C1—C2	−176.01 (12)	C8—N1—C7—C6	−175.09 (17)
Ni1—O1—C1—C6	3.6 (3)	Ni1—N1—C7—C6	4.4 (3)
O1—C1—C2—C3	179.49 (17)	C1—C6—C7—N1	−3.0 (3)
C6—C1—C2—C3	−0.1 (3)	C5—C6—C7—N1	177.10 (18)
C1—C2—C3—O2	−178.73 (16)	C7—N1—C8—C8^i^	171.2 (2)
C1—C2—C3—C4	1.4 (3)	Ni1—N1—C8—C8^i^	−8.3 (3)
O2—C3—C4—C5	179.35 (17)	C7—N1—C8—C9	−7.6 (3)
C2—C3—C4—C5	−0.8 (3)	Ni1—N1—C8—C9	172.86 (16)
C3—C4—C5—C6	−1.1 (3)	C8^i^—C8—C9—C10	−5.1 (3)
O1—C1—C6—C7	−1.2 (3)	N1—C8—C9—C10	173.65 (18)
C2—C1—C6—C7	178.37 (17)	C8—C9—C10—C10^i^	−1.6 (3)

Symmetry codes: (i) −*x*, *y*, −*z*+3/2; (ii) −*x*+1/2, −*y*+1/2, −*z*+2; (iii) *x*+1/2, −*y*+1/2, *z*−1/2; (iv) *x*−1/2, −*y*+1/2, *z*−3/2; (v) −*x*, *y*, −*z*+5/2.

### Hydrogen-bond geometry (Å, °)

**Table:**

*D*—H···*A*	*D*—H	H···*A*	*D*···*A*	*D*—H···*A*
O1W—H1W1···O1	0.88	2.40	3.0733 (18)	133
O1W—H1W1···O1^i^	0.88	1.97	2.8072 (19)	160
O1W—H2W1···O2^vi^	0.83	2.17	2.9985 (19)	173
C9—H9A···O2^vii^	0.93	2.60	3.394 (2)	144

Symmetry codes: (i) −*x*, *y*, −*z*+3/2; (vi) *x*, *y*, *z*−1; (vii) −*x*+1/2, *y*−1/2, −*z*+5/2.
